# Ser341Pro *MYOC* gene mutation in a family with primary open-angle glaucoma

**DOI:** 10.3892/ijmm.2015.2138

**Published:** 2015-03-13

**Authors:** FENGYUN WANG, YANG LI, LAN LAN, BO LI, LI LIN, XIAOHE LU, JIPING LI

**Affiliations:** 1 Department of Ophthalmology, The First Affiliated Hospital, Henan University of Science and Technology, Luoyang, Henan 471003; 2 Beijing Institute of Ophthalmology, Beijing Tongren Eye Center, Beijing Tongren Hospital, Capital Medical University, Beijing Ophthalmology and Visual Science Key Laboratory, Beijing 100730; 3 Department of Ophthalmology, Zhujing Hospital, Nanfang Medical University, Guangzhou, Guangdong 510515, P.R. China

**Keywords:** myocilin, primary open-angle glaucoma, mutation, genotype, phenotype

## Abstract

Glaucoma is known to induce visual impairment and blindness. The aim of the present study was to determine the clinical and genetic findings of a family with primary open-angle glaucoma (POAG). A family diagnosed with glaucoma was examined clinically and followed up for five years. Genomic DNA was extracted from the venous blood of 12 family members, and of 100 healthy individuals. The mode of inheritance was determined by the pedigree analysis. The third exon and its flanking introns of myocilin *(MYOC)* were amplified, and quantitative polymerase chain reaction (qPCR) products were sequenced. The restriction fragment length polymorphism analysis was performed on samples from the 12 family members and 100 normal controls. The predicted effects of the detected variants on the secondary structure of the MYOC protein were analyzed by the Garnier-Osguthorpe-Robson method. In this family, three members were diagnosed with POAG, and one member with ocular hypertension. The mode of inheritance of the family was autosomal dominant with six members being genetically affected. The heterozygous mutation was identified in the third exon of *MYOC* that revealed a T→C transition at position 1021 (p.S341P), which switched serine (Ser) to proline (Pro). This is a missense mutation eliminating a *CviKI-1* restriction site that segregated the affected members. Secondary structure prediction of p.S341P suggested that the MYOC protein was misfolded. Ser341Pro *MYOC* mutation was detected in the family with POAG. The clinical and genetic characteristics of this mutation require further investigation. The mutation spectrum of *MYOC* may be expanded for a better diagnosis and treatment for POAG patients.

## Introduction

Glaucoma is the second leading cause of visual impairment and blindness worldwide (1). It is a complex and chronic progressive optic nerve injury that is accompanied by high intraocular pressure (IOP), optic disc depression, optic nerve fiber atrophy and visual field defect. Primary open-angle glaucoma (POAG) is the most common type of glaucoma in developed countries, accounting for >50% of glaucoma cases (2). In China, the ratio of diagnosis with POAG has increased from 8.18% (1980s) to 19.25% (2012) in total glaucoma patients (3). A population-based cross-sectional study in China demonstrated that glaucoma was one of the major causes for blindness with POAG constituting the major type (4). In another study, it was found that the likelihood of developing POAG in relatives of POAG patients was 7- to 10-fold higher than that in the general population (5).

The pathogenesis of POAG is unknown. It is generally considered to be a multi-factorial disease and not a single genetic disorder with the single gene mutation. Epidemiological studies have demonstrated that genetic and environmental factors may affect its etiology, while genetic factors were mainly involved in the onset of POAG (6). Genetic linkage analysis of POAG pedigrees revealed that there were ≥20 genetic loci for POAG and only three causative genes were identified, i.e., myocilin (*MYOC*), optineurin (*OPTN*) and *WD* repeat domain 36 (*WDR36)* (7). Of the three genes, *MYOC* is deemed a direct causative gene leading to glaucoma, accounting for ~1 to 4% of mutations for POAG although the exact roles of *OPTN* and *WDR36* remain to be determined (7).

In the present study, we clinically followed up a Chinese POAG family over a period of five years. A pedigree analysis was performed, followed by molecular biology and bioinformatics analyses, which were used to investigate *MYOC* in the family members.

## Materials and methods

### Clinical data collection for study participants

This study was performed according to the tenets of the Declaration of Helsinki for Research Involving Human Subjects and was approved by the Ethics Committee of the First Affiliated Hospital at Henan University of Science and Technology (Henan, China). The glaucoma family had five generations of 29 members. Twelve members from three generations participated in this study and were numbered from 096001 to 096012. Informed consent was obtained from the 12 family members and 100 healthy controls.

The 12 family members received ophthalmologic examination including visual acuity, the anterior chamber, IOP measurement by applanation tonometry (Goldmann), anterior chamber angle evaluation by gonioscopy (Goldmann), fundus examination with a 90-diopter VOLK lens, and Octopus perimeter examination. The family members were clinically followed up over a period of five years, from 2005 to 2010. Diagnosis of POAG was based on the observation of at least two of the following abnormalities: characteristic glaucomatous optic disc changes [vertical cup-disc (c/d) ratio of ≥0.7, notching of the neutral rim, and disc hemorrhage], characteristic glaucomatous visual field defects, and high IOP (>21 mmHg) in the presence of a normal open anterior chamber angle. Diagnosis was made after the other secondary glaucoma was excluded, such as traumatic, uveitis, steroid-induced, and neovascular glaucoma. The age of diagnosis of the patients with POAG <35 years old was sub-classified as juvenile-onset open-angle glaucoma (JOAG). Individuals with IOP >22 mmHg but with no characteristic optic disc damage or visual field impairment were defined as ocular hypertension (OHT). Unaffected individuals had IOP in the normal range and optic nerves presented normal in appearance. In total, 100 healthy individuals (42 males and 58 females, 59.8±20.5 years) were included in the control group. A comprehensive eye examination was conducted for the healthy controls to exclude glaucoma and other genetic diseases including diabetes, blood hypertension, retinoblastoma, and high myopia.

### Genomic DNA collection

Genomic DNA was extracted from the venous blood of 12 family members and 100 healthy controls. Peripheral blood of 5 ml was collected from all the participants. Genomic DNA was extracted following the standard phenol/chloroform extraction method.

### Primer design and synthesis

According to the *MYOC* sequence, published by the USA National Center for Biotechnology Information, primers were designed to target the third Exon using Primer3 software (Table I) and produced by Beijing SBS Genetech Co., Ltd. (Beijing, China).

### Polymerase chain reaction (PCR)

The reaction (20 *μ*l) mix contained 2 *μ*l 10X PCR reaction buffer (20 mM Mg^2+^), 0.5 *μ*l dNTPs (10 mmol/l), 0.5 *μ*l forward and reverse primers (each 10 pmol/*μ*l), 0.5 *μ*l *Taq*DNA polymerize (2 U/*μ*l), 2 *μ*l template genome DNA (5–50 ng/*μ*l) and 14 *μ*l double-distilled water. The reaction conditions used were : i) 94°C pre-degeneration for 5 min; ii) 94°C degeneration for 30 sec; annealing for 30–60 sec; extension at 72°C for 30–60 sec; total circulation of 30- to 36-fold; iii) extension at 72°C for 10 min, and 1% agarose gel electrophoresis to detect the amplification effect.

### Purification, sequencing and comparison

The amplified PCR products and corresponding primers were purified and sequenced. An automatic fluorescence DNA sequencer (ABI PRISM 373A; Perkin-Elmer, Foster City, CA, USA) was used to sequence the purified PCR products in the forward and reverse directions. The sequencing results were compared with the published DNA sequence of *MYOC* (GenBank NM_000261) to screen mutations.

### Restriction fragment length polymorphism (RFLP) analysis

To confirm the variations found in the sequencing, restriction endonuclease CviKI-1 (New England Biolabs, Ipswich, MA, USA) was used for all the participants. The reaction was performed in a 10 *μ*l volume containing 9.4 *μ*l PCR product, 0.1 *μ*l BSA (100 *μ*g/ml), and 0.5 *μ*l enzyme (10 U/*μ*l). After incubating the reaction overnight at 37°C, the entire digest was run on a 1% agarose gel (with EB) at 100 V for 40 min and visualized under ultraviolet light.

### Bioinformatics analysis

Garnier-Osguthorpe-Robson (GOR) software was used to predict the effect of the mutation on the secondary structure of *MYOC*. It infers the secondary structure of a sequence by calculating the probability for each of the four structure classes (helix, sheet, turn and loop) based on the central residue and its neighbors from the calculated matrices.

## Results

### Pedigree structure of the POAG family

**Figure 1:**
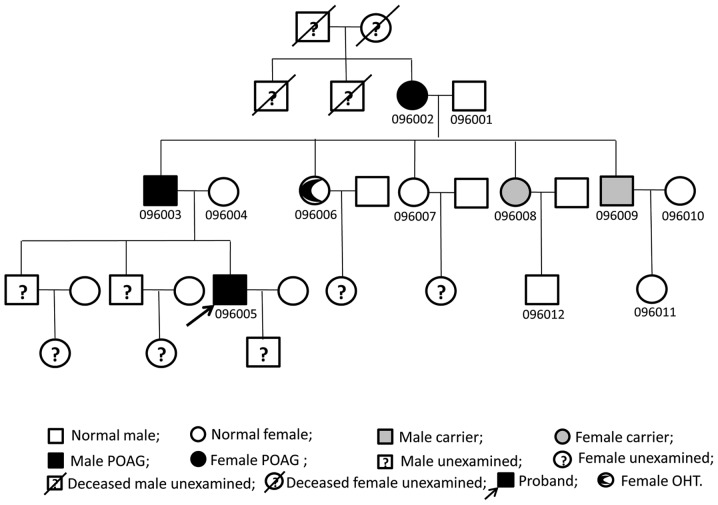
Pedigree structure of the primary open-angle glaucoma (POAG) family.

The family comprised 29 members of five generations. Twelve members from three generations participated in the study, and POAG was diagnosed in three members from each generation. POAG affected males and females in this family. The disease was passed vertically from generation to generation and was not gender-specific, indicating autosomal dominant genetic characteristics (Fig. 1).

### Clinical manifestations

**Figure 2:**
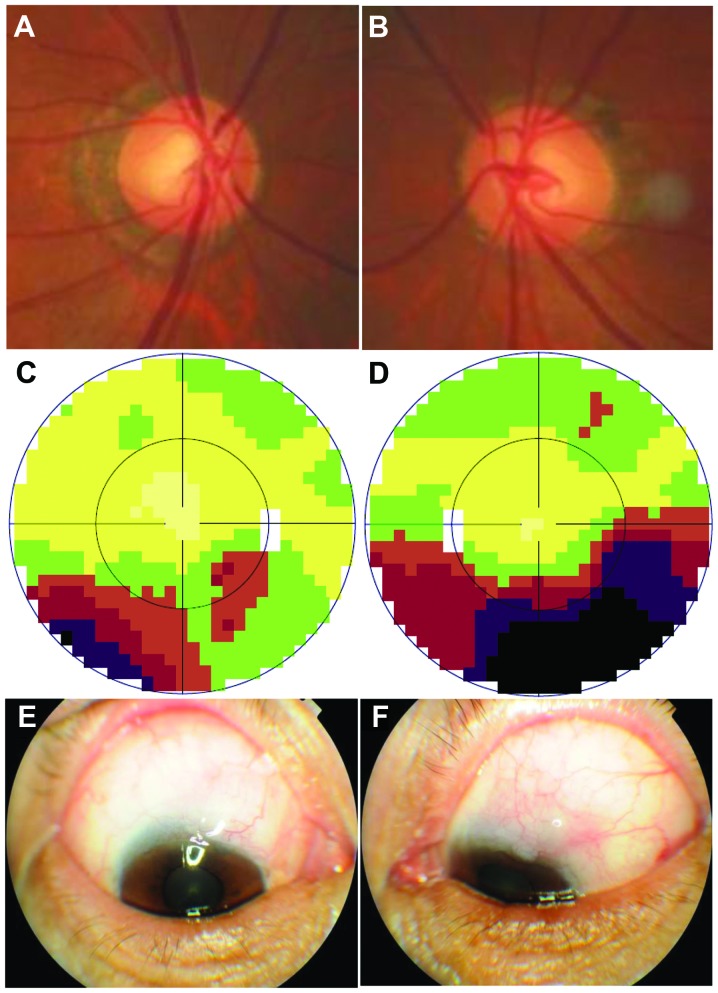
Images were obtained from one primary open-angle glaucoma (POAG) patient (096003) in the family. (A) Fundus image of the right eye, the C/D ratio is ~0.6, and the myopia arc is visible. (B) Fundus photograph taken from the left eye, the C/D ratio is ~0.7, and the myopia arc is also visible. (C) The map of the visual field (Octopus) of the right eye. The lower part of the visual field was defective, shown as brown, red and purple on the map. (D) The map of the visual field of the left eye. The lower half of the visual field was defective, shown as brown, red, purple and black on the map. The defect was more severe than that of the right eye. (E and F) Functional conjunctival blebs were observed in a follow-up examination following trabecule ctomy. Conjunctival hyperemia was not apparent.

Three members (096002, 096003 and 096005) were diagnosed with POAG, and another one (096006) was diagnosed with OHT in this family (Table II). The proband of the family was diagnosed at 25 years of age, which was consistent with the diagnosis of JOAG. Visual acuity, IOP, optic nerve, and visual field were affected most severely among the three patients. The other two POAG patients were all diagnosed at >50 years of age and the exact age of onset was not known. The member who was diagnosed with OHT was 55 years old at the last visit. She had a maximum IOP 21/24 and a borderline C/D ratio (0.6/0.6) without the visual field defect. Trabeculectomy was performed on three POAG patients. Post-operative IOP is shown in Table II and two of the patients (096003 and 096005) required anti-glaucoma eye drops even though the filtering blebs were functional (Fig. 2). The remaining family members were treated as normal according to the diagnostic criteria.

### Sequencing and comparative analysis

**Figure 3:**
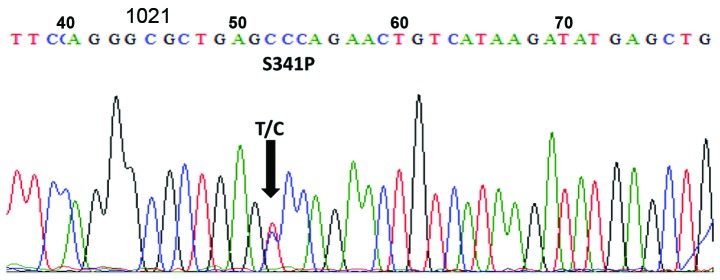
DNA sequence chromatograms. The sequence of the third exon of myocilin (*MYOC*) is highlighted. Heterozygote sequence (sense strand) shows a T/C transition in codon 341 that changed serine (TCC) to proline (CCC).

The third exon of *MYOC* was PCR amplified and received direct sequencing in the 12 family members. We found c.1021 T→C heterozygous base change (Fig. 3) in members 096002, 096003, 096005, 096006, 096008 and 096009. The codon changed from TCC to CCC, thus amino acids at position 341 were altered. Consequently, the corresponding myocilin protein was altered from serine (Ser) into proline (Pro) (p.S341P). This mutation was not identified in members 096001, 096004, 096007, 096010, 096011 and 096012, or the 100 normal controls.

### Restriction fragment length polymorphism

**Figure 4:**
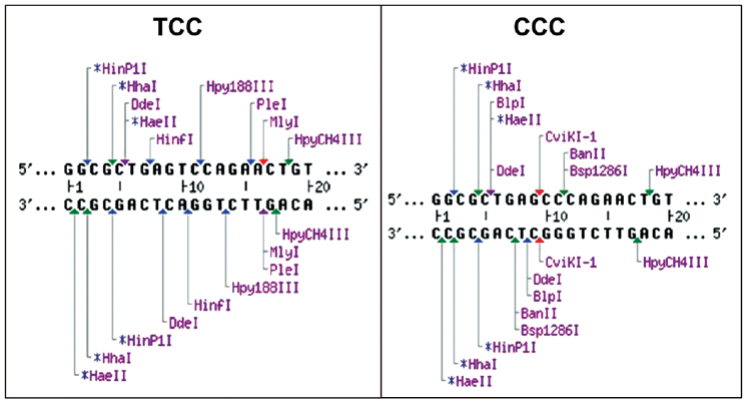
Restriction endonuclease recognition sites. The DNA sequences, enzyme loci and restriction fragment size. The left panel shows the normal sequence of the third exon of myocilin (*MYOC*). The right panel shows the sequence with a T/C transition in codon 341 which changed serine (TCC) to proline (CCC), and the mutation caused the loss of restriction sites of *CviKI-1* enzyme in the corresponding DNA sequence.

**Figure 5:**
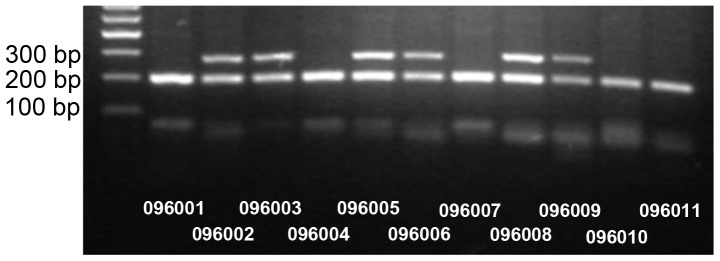
Restriction fragment length analysis showed that the p.S341P mutation abolished a CviKI-1 site co-segregated with POAG patients (096002, 096003 and 096005), ocular hypertension (096006), and carriers (096008 and 096009), but not with unaffected individuals (096001, 096004, 096007, 096010, 096011 and 096012).

Based on the results of *MYOC* sequencing, we designed the relevant restriction endonuclease sites. We found that *CviKI-1* can recognize c.DNA1020 on base G and c.DNA1021 on base T in the normal *MYOC*. The c.1021T→C (p.S341P) mutation caused loss of the restriction enzyme sites of *CviKI-1* in the corresponding DNA sequence. In Table III and Fig. 4, we show the restriction endonuclease recognition sequence, enzyme loci and restriction fragment size. Loss of *CviKI-1* sites was found in three POAG patients and one OHT patient, which was also identified in two members (096008 and 096009) who were not clinically diagnosed with POAG (Table II). The homologous chromosomes of family members (096002, 096003, 096005, 096006, 096008 and 096009) who had the mutation were cleaved into four fragments (257, 209, 203 and 48 bp). The homologous chromosomes of other members (096001, 096004, 096007, 096010 and 096011) were cleaved into three fragments (209, 203 and 48 bp) (Fig. 5). The three members (096006, 096008 and 096009) without POAG diagnosis at the last visit were considered to be gene mutation carriers.

### Protein secondary structure prediction

**Figure 6:**
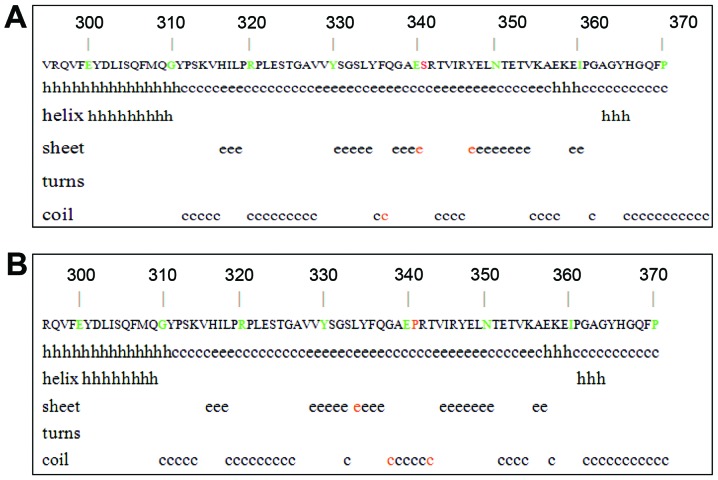
The effect of p.S341P on the secondary structure of myocilin protein. (A) The secondary structure of the wild-type myocilin protein around the site S341. (B) The secondary structure of the mutant P341 of the myocilin protein in the corresponding region. Red letters shows the positions of change.

The *MYOC* c.1021T→C base change caused amino acid switch from Ser to Pro. We found that changes at sites 333, 337 and 342. The coil structure of amino acids 333 was replaced with the β-sheet structure, and the β-sheet structure of amino acids 337 and 342 were replaced with the coil structure (Fig. 6). The base change was at the junction of the coil structure and the lamellar structure.

### Homology analysis of protein

**Figure 7:**
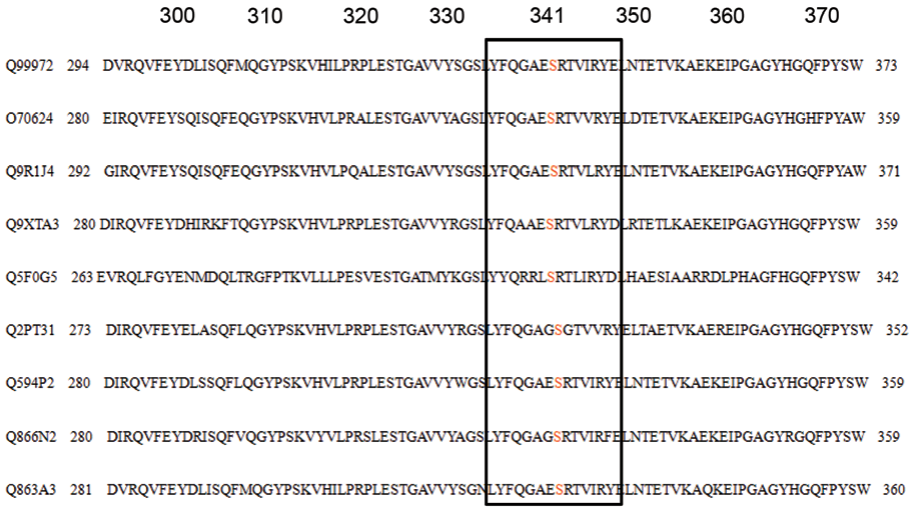
Sequence alignment portion of the olfactomedin-like domain spanning the missense mutation p.S341P of human myocilin (MYOC) and a comparison with the other species.

The homology analysis was applied to compare οlfactomedin-like domains of amino acid coding of human (Q99972) with other mammals (rat (Q9R1J4), mouse (O70624), cattle (Q9XTA3), dog (Q2PT31), rabbit (Q866N2), cynomolgus (Q863A3), cat (Q594P2), and zebrafish (Q5F0G5)). We found that amino acid 341 was conserved as Ser (Fig. 7).

## Discussion

*MYOC*, also known as the *TIGR* (trabecular meshwork inducible glucocorticoid response) gene, is the first causative gene identified in JOAG. It contains three exons, which are 604, 126 and 782 base pairs respectively. The product, myocilin protein, is composed of 504 amino acids (8). The N-terminus of myocilin protein has a signal peptide sequence, a myosin-like domain, and a zinc finger-like domain. The C-terminal of myocilin protein has an olfactory factor (Olfactomedin) domain. The *MYOC* promoter region contains a number of important gene regulation motifs (9). The POAG pathogenesis was associated with at ≥70 *MYOC* mutation sites (10), with ~90% in the third exon of the οlfactomedin homologous region (11). This domain is the active site of protein function as that οlfactomedin homologous has an important role for the function of myocilin protein expression.

**Figure 8:**
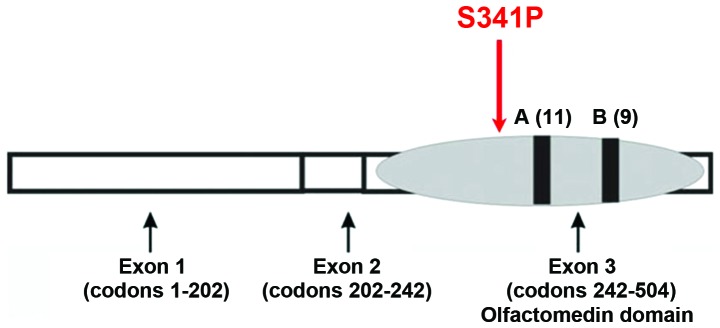
Schematic diagram of the Ser341Pro myocilin (*MYOC*) mutation.

In the present study, the *MYOC* mutation was located in οlfactomedin homology and this mutation was not found in normal conditions (Fig. 8). This mutation was a heterozygosity mutation and switched Ser to Pro. The two amino acids possess different physical and chemical properties. Ser is a mixed hydrophilic amino acid with a molecular weight of 105.09 and an isoelectric point of 5.68. The relative distance of Ser is 2,000; the irreplaceable nature is 0.64 and the number of codons is six. By contrast, Pro is a highly hydrophilic amino acid with a molecular weight of 115.13 and an isoelectric point of 6.30. The relative distance of Pro is 1,720; the irreplaceable nature is 0.61 and the number of codons is four (12).

Our homology analysis showed that amino acid 341 is Ser across various species. Prediction of the protein secondary structure suggested that myocilin protein may misfold and become hydrophobic with lower solubility, which blocks the trabecular meshwork and affects the outflow of aqueous humor. It is known that there are 13 *MYOC* mutations for POAG in China: P13L (13), Q337Stop (13), S341P (14), P370L (10,15–17), C245Y (18,19), T353I (19,20), R91X(20), G252R (15,21), E300K (20,19), S313F (19), N450Y (23), Y471C (20,19), and T455K (22). These mutations were identified only in Chinese patients with POAG, suggesting *MYOC* mutation had ethnic and regional specificity. Although genes in the Chinese POAG patients have been studied extensively (13,14,16–18,20–27), additional investigations are necessary to provide evidence for the pathogenesis of POAG for treatment and development of drugs (28–30).

Incomplete penetrance has been observed in most families with MYOC mutations and the penetrances are age-dependent and mutation-specific (10,15,31). The pedigree analysis revealed two clinically healthy individuals and one OHT patient who harbored the mutation of p.S341P and the affected haplotype. The penetrance of this pedigree was that 66.67% (4/6) of the individuals carrying the p.S341P mutation had developed POAG or OHT. With the exception of the proband of the family, who was 25 years of age at diagnosis, the remaining patients were >50 years of age at diagnosis and their symptoms were less severe than those of the proband. This observation suggests that unidentified factors (genetic or environmental) may be associated with the POAG of this pedigree. However, carriers should undergo ophthalmologic surveillance at regular intervals.

The Ser341Pro mutation was detected in our study and further investigation of the novel p.S341P mutation of *MYOC* for POAG was necessary. The mutation spectrum of *MYOC* may be expanded and a better diagnosis and treatment for POAG patients may be achieved in the future.

## Figures and Tables

**Table I:** Primers of the third exon in the *MYOC* gene.

Exon	Primer	Primer sequence (5′–3′)	Product size (bp)	Optimal annealing temperature (°C)
3	MYOC-3-1F	CTTCCGCATGATCATTGT	352	58
	MYOC-3-1R	CTTCCGCATGATCATTGT		
3	MYOC-3-2F	ATACTGCCTAGGCCACTGGAA	440	58
	MYOC-3-2R	CCGCTATAAGTACAGCAGCATGAT		
3	MYOC-3-3F	GCCTTCATCATCTGTGGCAC	342	58
	MYOC-3-3R	CAGGCAGCTTTGACTGCTTT		

F, forward; R, reverse; MYOC, myocilin.

**Table II:** Clinical parameters of individuals in this pedigree.

Pedigree ID no.	Gender/age (year)	Diagnosed at age (year)	BCVA OD/OS (2005)	BCVA OD/OS (2010)	Maximum IOP OD/OS mmHg	IOP (2005) OD/OS mmHg	IOP (2010) OD/OS mmHg	Optic Disc (C/D) OD/OS (2010)	VF Loss OD/OS	Therapy OD/OS	Diagnosis 2005	Diagnosis 2010	S341P
096001	M/82		0.6/0.5	0.4/0.25	17/17	17/17	16/16	0.3/0.4	No/No		Normal	Normal	No
096002	F/82	65	0.5/0.5	0.3/0.2	17/22	17/18	17/22	0.4/0.5	Yes/Yes	S/S	POAG	POAG	Yes
096003	M/61	55	1.0/0.8	0.6/0.6	52/50	45/40	30/30	0.6/0.7	Yes/Yes	s/s	POAG	POAG	Yes
096004	F/60		1.0/1.0	1.0/0.8	17/17	17/17	17/17	0.3/0.4	No/No		Normal	Normal	No
096005	M/32	25	1.2/CF	1.0/CF	48/NA	48/NA	17/33	0.8/0.9	Yes/Yes	S/S	POAG	POAG	Yes
099006	F/55	50	1.0/1.0	1.0/1.0	21/24	17/20	21/24	0.6/0.6	No/No	NMT	OHT	OHT	Yes
096007	F/49		1.2/1.2	1.0/1.0	17/16	17/17	17/16	0.3/0.3	No/No		Normal	Normal	No
096008	F/44		1.0/1.2	1.0/1.0	17/18	17/18	17/18	0.5/0.4	No/No		Normal	carrier	Yes
096009	M/41		1.0/1.2	1.0/1.2	17/21	17/21	17/21	0.4/0.4	No/No		Normal	carrier	Yes
096010	F/40		1.0/1.2	1.0/1.2	17/17	16/16	16/16	0.3/0.3	No/No		Normal	Normal	No
096011	F/14		1.0/1.0	1.0/1.2	16/16	16/16	16/16	0.3/0.3	No/No		Normal	Normal	No
096012	M/20		1.0/1.0	1.0/1.0	17/17	NA	NA	0.3/0.3	No/No		Normal	Normal	No

M, male; F, female; BCVA, best-correct visual acuity; OD, right eye; OS, left eye; CF; count fingers; IOP, intraocular pressure; C/D, cup disc ratio; VF, visual field; NMT, no medical therapy; NA, unavailable; S, surgery; OHT, ocular hypertension.

**Table III:** The CviKI-1 endonuclease recognition sequence, number of restriction sites and size of fragment on RFLP analysis.

*MYOC* exon (mutation)	Endonuclease	Recognition sequence	Number of restriction	Size of fragment (bp)
Exon 3 (c.1021T→C)	*CviKI-1*	5′…GAG▾CCC---3′ 3′…CTC▾GGG---5′	1	257 (209/203/48)

RFLP, restriction fragment length polymorphism; MYOC, myocilin.
